# Global Analysis of Neutrophil Responses to *Neisseria gonorrhoeae* Reveals a Self-Propagating Inflammatory Program

**DOI:** 10.1371/journal.ppat.1004341

**Published:** 2014-09-04

**Authors:** Anna Sintsova, Helen Sarantis, Epshita A. Islam, Chun Xiang Sun, Mohsen Amin, Carlos H. F. Chan, Clifford P. Stanners, Michael Glogauer, Scott D. Gray-Owen

**Affiliations:** 1 Department of Molecular Genetics, University of Toronto, Toronto, Ontario, Canada; 2 Faculty of Dentistry, University of Toronto, Toronto, Ontario, Canada; 3 Department of Biochemistry and Goodman Cancer Research Centre, McGill University, Montreal, Quebec, Canada; University of California, Davis, United States of America

## Abstract

An overwhelming neutrophil-driven response causes both acute symptoms and the lasting sequelae that result from infection with *Neisseria gonorrhoeae*. Neutrophils undergo an aggressive opsonin-independent response to *N. gonorrhoeae*, driven by the innate decoy receptor CEACAM3. CEACAM3 is exclusively expressed by human neutrophils, and drives a potent binding, phagocytic engulfment and oxidative killing of Opa-expressing bacteria. In this study, we sought to explore the contribution of neutrophils to the pathogenic inflammatory process that typifies gonorrhea. Genome-wide microarray and biochemical profiling of gonococcal-infected neutrophils revealed that CEACAM3 engagement triggers a Syk-, PKCδ- and Tak1-dependent signaling cascade that results in the activation of an NF-κB-dependent transcriptional response, with consequent production of pro-inflammatory cytokines. Using an *in vivo* model of *N. gonorrhoeae* infection, we show that human CEACAM-expressing neutrophils have heightened migration toward the site of the infection where they may be further activated upon Opa-dependent binding. Together, this study establishes that the role of CEACAM3 is not restricted to the direct opsonin-independent killing by neutrophils, since it also drives the vigorous inflammatory response that typifies gonorrhea. By carrying the potential to mobilize increasing numbers of neutrophils, CEACAM3 thereby represents the tipping point between protective and pathogenic outcomes of *N. gonorrhoeae* infection.

## Author Summary

Gonorrhea is a sexually transmitted infection caused by the bacteria *Neisseria gonorrhoeae*. These bacteria have re-emerged as a public health priority due to its acquisition of resistance to multiple antibiotics, leading to fears of untreatable infection. The symptoms of gonorrhea include an intense inflammatory response that may lead to pus discharged from the infected genital tract and scarring of the reproductive tract caused by neutrophils recruited to the site of infection. Past studies have detailed molecular interactions that lead to neutrophil binding and engulfment of *N. gonorrhoeae*, yet it remains unclear why *N. gonorrhoeae* elicits such a pathogenic inflammatory response. In this study, we reveal that *N. gonorrhoeae* binding to the human innate decoy receptor, CEACAM3, elicits a potent intracellular signaling cascade that leads to neutrophil expression of cytokines that actively recruit other neutrophils to the infected tissues. As they encounter the gonococci, the next wave of neutrophils becomes similarly activated, leading to the progressive expansion in phagocytic cell numbers until they overwhelm the infected tissues. While this process promotes a rapid response to a troubling pathogen early during infection, the unrestrained recruitment of neutrophils and their toxic antimicrobial arsenal also lead to the pathogenic consequences associated with gonorrhea.

## Introduction

*Neisseria gonorrhoeae*, the causative agent of gonorrhea, is a re-emerging global health concern, with over a hundred million cases diagnosed each year, the recent emergence of multi-drug resistant strains that have led to its ‘superbug’ status, and a lack of success in vaccine development [1], [2], [3]. Symptomatic infection with *N. gonorrhoeae* results in acute inflammation of the urogenital tract and a purulent urethral discharge consisting almost exclusively of neutrophils. If left untreated, gonococcal infection can lead to serious chronic conditions, such as pelvic inflammatory disease and infertility, which stem from an overzealous response to the infection [2].

*N. gonorrhoeae* is a Gram-negative diplococcus that is highly adapted to colonization of the human urogenital tract. The initial interaction between the bacteria and epithelia is mediated by type IV pili, which retract to allow tight association with the mucosal epithelia [4]. More intimate interactions are then facilitated by adhesins including the neisserial Opa proteins binding to certain epithelial cell-expressed members of the carcinoembryonic antigen-related adhesion molecule (CEACAM) family: CEACAM1, CEACAM5, and CEACAM6 [5], [6], [7], [8], [9]. CEACAMs represent a subset of the Ig superfamily and consist of a variable number of Ig-like constant domains and an Ig variable domain-like N-terminus that allows Opa binding [10], [11], [12]. Attachment to apically expressed CEACAMs is sufficient to trigger bacterial engulfment and transcytosis across the epithelia to allow entry into the subepithelial space [13], [14]. CEACAM1 is notable among the family in that, in addition to being on epithelial cells, it is also expressed on certain endothelial, lymphocytic and myeloid cells. Bacteria exploit its co-inhibitory function, which depends upon its cytoplasmic immunoreceptor tyrosine-based inhibitory motif (ITIM), to suppress T cell [15], [16], [17], B cell [18], dendritic cell [19] and epithelial cell [20] responses (reviewed in [21]).

While binding to CEACAMs on most cell types tends to facilitate infection, Opa proteins may also bind to neutrophil-expressed CEACAM3. When this occurs, CEACAM3 triggers an efficient opsonin-independent phagocytosis of the bacteria [22], [23], [24]. Ligation of CEACAM3 also promotes a Syk kinase- and phosphatidylinositol 3-kinase-dependent recruitment and downstream activation of the neutrophils' antimicrobial responses, including degranulation and oxidative burst [22], [23], [24], [25], [26], [27], [28]. These effects are driven by the cytoplasmic immunoreceptor tyrosine-based activation motif (ITAM), which distinguishes CEACAM3 from the other CEACAMs that *N. gonorrhoeae* binds. Considering that CEACAM3 is human-restricted, expressed on neutrophils and lacks cell adhesion function, CEACAM3 is now generally considered to be an innate immune receptor allowing capture and elimination of bacteria that colonize epithelial tissues via other CEACAMs [23], [24], [29], [30], [31].

Neutrophils are specialized for rapid transmigration to sites of infection in response to a variety of stimuli, including chemotactic gradients and presence of bacterial components. Following recruitment to the infected tissue, neutrophils effectively phagocytose opsonized bacteria, activate production of reactive oxygen species [32] and release toxic antimicrobial peptides and proteins from cytoplasmic granules [33], [34]. Conventionally, neutrophils were thought to have little to no controlled expression of new gene products, depending mostly on constitutively-expressed proteins and pre-loaded granules assembled during maturation. In recent years, it has become evident that properly stimulated neutrophils respond by synthesizing new proteins [35], [36], [37], however surprisingly little is known about the control of gene expression.

In this work, we show that heterologous expression of human CEACAMs in transgenic mouse neutrophils permits effective opsonin-independent neisserial binding and neutrophil activation in a manner reflecting that seen with human neutrophils. Moreover, we reveal that Opa-dependent CEACAM3 binding drives a potent neutrophil transcriptional response that elicits production of pro-inflammatory cytokines via a PKCδ and Tak1 serine/threonine kinase-dependent pathway triggered downstream of Syk tyrosine kinase. Furthermore, we observed that infection of human CEACAM-expressing transgenic mice with *N. gonorrhoeae* results in a dramatically higher neutrophil influx to the infection site when compared to wild-type mice. Together, this study establishes that bacterial binding to CEACAM3 effectively recruits more neutrophils to the infected tissues. While providing an effective strategy to combat the initial infection, this self-propagating cycle of events can also lead to the pathogenic inflammatory response that typifies symptomatic gonorrhea.

## Results

### CEACAM-humanized transgenic mouse neutrophils respond to *N. gonorrhoeae*

**Figure 1:**
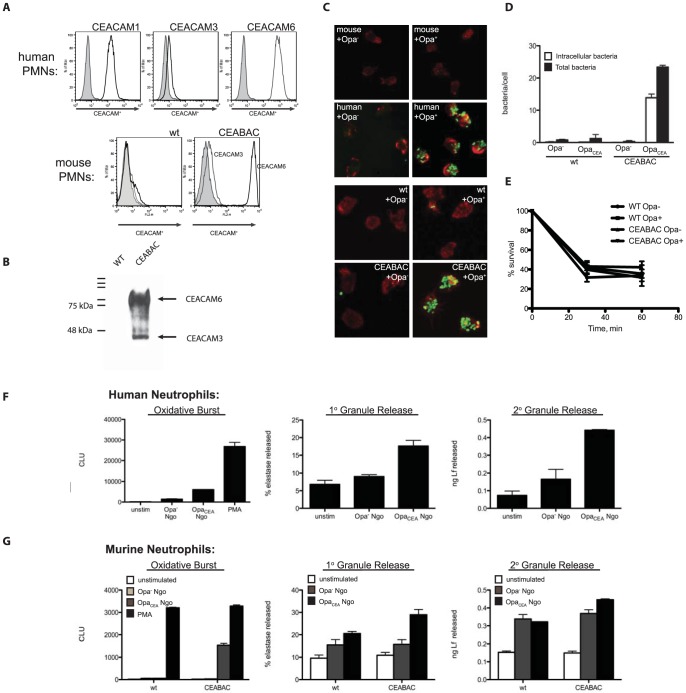
Human CEACAM expression by mouse neutrophils results in neisserial capture and internalization. (**A**) Human CEACAMs are expressed in CEABAC neutrophils in a manner reflecting that in human neutrophils. Human neutrophils (top), or wild type (WT) and CEABAC mouse neutrophils (bottom) were fixed and stained with antibodies specific for CEACAM1, CEACAM3, CEACAM6, or a mouse IgG isotype control, and analyzed by flow cytometry. Isotype histograms are shaded. (**B**) Immunoblot showing CEACAM expression in WT and CEABAC neutrophils. (**C**) Mouse neutrophils do not bind *N. gonorrhoeae*, while human neutrophils bind *N. gonorrhoeae* in an Opa-dependent manner. Mouse (top) or human (bottom) neutrophils were infected with non-opaque (Opa^−^) or Opa-expressing (Opa^+^) *N. gonorrhoeae*. Cells were visualized by staining filamentous actin with phalloidin [16], and bacteria are shown in green. Intracellular and total bacteria were differentially stained, and quantified via immunofluorescence microscopy (**D**). (**E**) WT and CEABAC PMNs kill Opa^−^ and Opa^+^ bacteria with similar kinetics. Adherent WT and CEABAC PMNs were infected with either Opa^−^ or Opa^+^ *N. gonorrhoeae* at an MOI = 1. Bacterial survival over time was evaluated as CFUs present in PMN lysates at each time point relative to bacterial CFUs present at time 0. N = 2. (**F–G**) *N. gonorrhoeae* infected CEABAC neutrophils respond analogously to human PMNs. Human (**F**) or mouse (WT and CEABAC) (**G**) neutrophils were infected with Opa^−^ or Opa^+^ *N. gonorrhoeae* and oxidative burst and degranulation responses were analyzed as described in Materials and Methods.

Neisserial infection is exquisitely human-specific, with major receptors for the bacterial Opa protein adhesins being certain members of the human CEACAM family. While CEACAM homologues can be found in all vertebrates [38], only human CEACAMs have been observed to bind *Neisseria*. Mouse polymorphonuclear leukocytes (PMNs), which express mouse CEACAM1 on their surface, do not bind *N. gonorrhoeae* [24], [39], whereas human PMNs specifically bind Opa-expressing but not Opa-deficient *N. gonorrhoeae* in an opsonin-independent fashion (Figure 1A, C). We have recently established that recombinant human CEACAMs encoded from constitutively expressed cDNA were functionally expressed in a mouse promyelocytic (MPRO) cell line [24]. We considered whether ectopic expression of intact human CEACAM genes in transgenic mouse neutrophils would also confer responsiveness to *N. gonorrhoeae*. To address this question, we performed experiments with bone marrow-derived neutrophils from human CEACAM-expressing CEABAC2 mice [40]. These mice were engineered using a BAC that encodes human CEACAM3, CEACAM5, CEACAM6 and CEACAM7, none of which have murine homologues. Using flow cytometric analysis and immunoblotting with CEACAM-specific antibodies, we confirmed that human CEACAM3 and CEACAM6 were expressed on the surface of CEABAC neutrophils, in a manner reflecting their expression on human neutrophils (Figure 1A,B). When we exposed CEABAC neutrophils to *N. gonorrhoeae* expressing either the CEACAM-specific Opa^+^ or no Opa protein (Opa^−^), they effectively bound and engulfed the Opa^+^ but not Opa^−^ bacteria, whereas no such association was apparent with wild type neutrophils regardless of Opa expression (Figure 1C,D). While the number of Opa-expressing bacteria captured by human CEACAM-expressing neutrophils was substantially (∼10-fold) greater than what occurs with WT mouse neutrophils and/or Opa^−^ bacteria (Figure 1D), the bacteria that become engulfed are effectively killed regardless of whether or not human CEACAMs are involved in the uptake (Figure 1E). These results differ from a recent study with human neutrophils which describe increased killing of Opa^+^ (relative to Opa^−^) bacteria [41], however it remains unclear whether this is a neutrophil species-dependent effect or result from differences in bacterial strain or methodology used in the two studies.

### *N. gonorrhoeae* infection activates CEABAC neutrophils

Previous work addressing individual CEACAM expression and its effect on neisserial infection was undertaken using transfected promyelocytic cell lines [24]. Because CEABAC transgenic neutrophils can bind and engulf *N. gonorrhoeae* (Figure 1C), we wondered whether neutrophil-specific responses to *N. gonorrhoeae* were also reproduced in these cells. Human neutrophils respond to Opa-expressing *N. gonorrhoeae* by triggering an increased consumption of oxygen, resulting in the production of free oxygen radicals in the cell (the ‘oxidative burst’), as well as by releasing granule components to the cell surface or into the newly formed phagosome (‘degranulation’) [25]. Consistent with this, we observed that Opa-expressing *N. gonorrhoeae* were able to efficiently stimulate both the oxidative burst and release of primary and secondary granules (as determined by the release of neutrophil elastase and lactoferrin, respectively), in infected human neutrophils (Figure 1F). In stark contrast, WT mouse neutrophils are surprisingly unresponsive to *N. gonorrhoeae* infection, illustrating the importance of human CEACAMs for these effects. However, in CEABAC neutrophils, we observed that the oxidative burst and degranulation were heightened in response to Opa-expressing bacteria (Figure 1G), consistent with the function of Opa proteins in CEACAM binding.

### *N. gonorrhoeae* drives an acute inflammatory program in neutrophils

**Figure 2:**
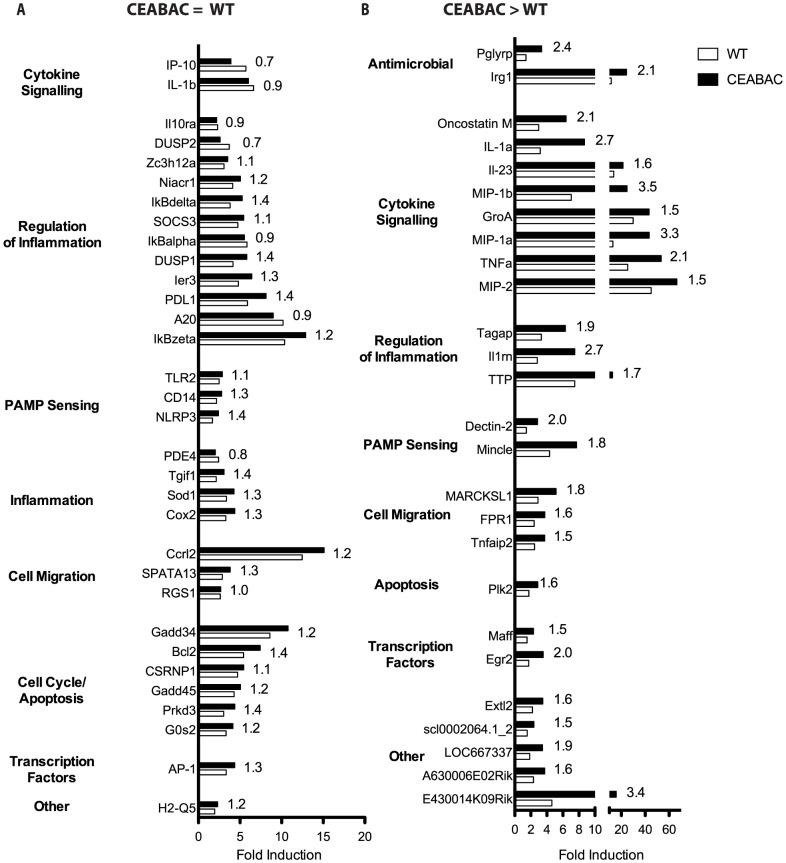
*N. gonorrhoeae* drives an acute inflammatory transcriptional program. A gene array of WT and CEABAC PMNs infected with Opa^+^ *N. gonorrhoeae* was performed, and genes showing a significant increase in transcription over uninfected controls are illustrated. Genes with ≥2 fold induction over uninfected controls in both WT and CEABAC neutrophils 1 h post-infection are shown. (**A**) List of genes up-regulated to a similar level in both WT and CEABAC neutrophils relative to uninfected neutrophils. (**B**) List of genes differentially expressed between CEABAC and WT PMNs. The fold difference by which WT and CEABAC differ is indicated next to each bar.

While neutrophils were classically considered to be transcriptionally quiet, Fc receptor-mediated phagocytosis has long been known to promote IL-8 mRNA expression in neutrophils [32]. More recently, it has become clear that neutrophils have the capacity to become transcriptionally active in response to certain stimuli [35], [36], [37], yet neutrophil transcriptional responses to specific infections remain poorly understood. Consequently, to investigate whether *N. gonorrhoeae* might elicit a transcriptional response, we isolated RNA from uninfected and infected WT and CEABAC bone marrow-derived neutrophils and compared their transcriptional profile by full genome gene array 1 hour post-infection. DAVID functional annotation [42] and manual analysis of the results revealed that the general pattern of genes expressed in response to *N. gonorrhoeae* were similar in the WT and CEABAC animals, however, two categories of transcriptional up-regulation were apparent. In the first group are genes that are induced to a similar level in WT and CEABAC PMNs. Of these, the largest functional classes of genes are those involved in the regulation of inflammation (*i.e.* IL-10 Receptor α Subunit (il10ra); Suppressor of Cytokine Signaling 3 (SOCS3); Inhibitor of kappa B subunits (IκBδ, IκBζ)) and control of cell cycle and apoptosis (*i.e.* Bcl2, Gadd34, Gadd45) (Figure 2A). In contrast, the CEABAC neutrophils displayed a marked up-regulation of acute inflammatory cytokine expression, including TNFα (2.1-fold induction over wild type neutrophils), IL-1α (2.7-fold induction) and the neutrophil chemoattractant and activators Gro-α/KC, MIP-1α and MIP-1β (1.5-, 3.3- and 3.5-fold induction) (Figure 2B). It is pertinent to note that we detected no down-regulation of any gene expression at that time point. Considering that regulatory genes are expressed at similar levels between WT and CEABAC PMNs, while pro-inflammatory mediators are drastically higher in CEABAC cells, we infer that the cumulative effect of these changes in gene expression would be a heightened pro-inflammatory cytokine response when the gonococcal Opa proteins engage the neutrophil-expressed CEACAM3.

### Opa-expressing *N. gonorrhoeae* drive production of pro-inflammatory cytokines in CEACAM-expressing neutrophils

**Figure 3:**
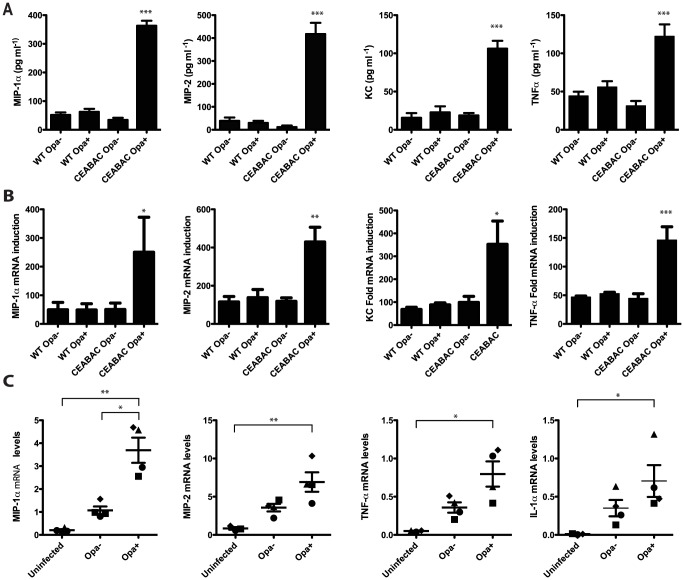
Opa-expressing *N. gonorrhoeae* drive CEACAM-dependent production of pro-inflammatory cytokines in neutrophils. (**A–B**) CEABAC PMNs secret pro-inflammatory cytokines in response Opa^+^ *N. gonorrhoeae*. Mouse (WT and CEABAC) neutrophils were infected with either Opa^−^ or Opa^+^ *N. gonorrhoeae* (MOI 10). MIP-1α, MIP-2, KC and TNFα production was measured 3 h post infection at (**A**) protein and (**B**) mRNA level. N≥3, error bars represent SEM. (**C**) Human neutrophils infected with Opa^+^ *N. gonorrhoeae* show increased levels of MIP-1α, MIP-2, TNFα, and IL-1α mRNA relative to PMNs infected with Opa^−^ bacteria. Each symbol represents an individual donor. Cytokine mRNA levels shown as relative to levels of GAPDH mRNA. One-Way ANOVA (with Tukey's post-test) was performed for relevant samples, *P<0.05, **P<0.01, ***P<0.001. For (**A**) and (**B**) stars indicate significance against all other conditions.

To validate gene array results, and confirm that increases in transcript levels corresponded to PMN secretion of the protein products, we measured the production of MIP-1α, MIP-2, KC and TNF-α protein in infected PMNs from WT and CEABAC mice (Figure 3A). Significant (between 3- to-10 fold) increases in chemokine protein levels were observed in supernatants from CEABAC PMNs infected with Opa^+^ bacteria, compared to supernatants from CEABAC PMNs infected with Opa^−^ bacteria. Critically, the WT neutrophil response was not affected by Opa expression, instead reflecting that seen with Opa^−^ bacteria and CEABAC PMNs, demonstrating that both Opa and human CEACAMs are required for this effect. This increased chemokine secretion corresponded with increased levels of chemokine transcripts in these samples (Figure 3B), reflecting the data obtained via the gene array experiments, and establishing that *de novo* transcription is driving the cytokine response. Collectively, these data provide the first evidence of a neutrophil transcription-based inflammatory response to *N. gonorrhoeae* infection, and point to the CEACAM-Opa interaction as a critical driver of this inflammation.

### CEACAM-Opa interaction drives an inflammatory response in human peripheral blood neutrophils

To confirm that the CEACAM- and Opa-dependent transcriptional response apparent in our transgenic mouse model reflected that occurring in human PMNs, peripheral blood neutrophils isolated from healthy volunteers were infected with Opa^−^ or Opa^+^ *N. gonorrhoeae* and then subjected to quantitative RNA analysis. Opa^+^ *N. gonorrhoeae*-infected PMNs had substantially higher levels of MIP-1α, MIP-2, TNF-α, and IL-1α transcript in all donors tested, when compared to Opa^−^ infected controls (Figure 3C). We therefore conclude that the CEACAM-Opa interaction potentiates the inflammation observed during human PMN infection, reflecting our findings with the CEACAM-humanized mouse model.

### Phagocytosis and production of reactive oxygen species are not essential for inflammatory cytokine production

**Figure 4:**
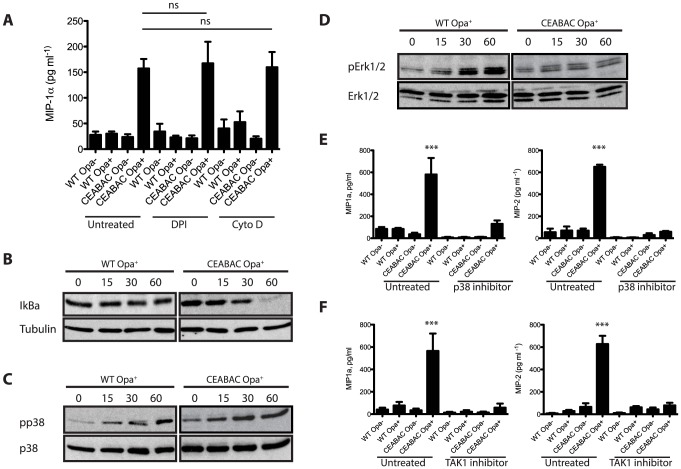
*N. gonorrhoeae* infection elicits an NF-κB and p38 MAPK signaling-dependent cytokine response in CEABAC neutrophils. CEABAC-Opa interaction leads to activation of NF-κB signaling. (**A**) WT and CEABAC PMNs were left untreated or pre-incubated with DPI (10 µM) or cytochalasin D (10 mg/ml). PMNs were then infected with either Opa^−^ or Opa^+^ *N. gonorrhoeae* and MIP-1α levels were measured 3 h post infection. N = 3, error bars represent SEM. One-Way ANOVA indicates no significant differences between corresponding samples. (**B**) WT and CEABAC PMNs were infected with Opa^+^ *N. gonorrhoeae* (MOI = 10 bacteria/PMN) for times indicated, and levels of IkBα were then detected by immunoblot with IκBα antibody. Assessment of tubulin levels confirmed equal protein loading. (**C, D**) CEABAC-Opa interaction promotes activation of p38 MAPK. WT and CEABAC PMNS were infected as in (**B**). Activation of MAP kinases p38, Erk1, and Erk2 was determined by immunoblot with phospho-specific antibodies. Total p38 and Erk1/2 levels were assessed to ensure equal protein loading. (**D, F**) WT and CEABAC PMNs were left untreated or preincubated with (**D**) p38 inhibitor (SB203580, 10 µM) or (**E**) TAK1 inhibitor ((5z)-7-oxozeaenol, 500 nM). PMNs were then infected with either Opa^−^ or Opa^+^ *N. gonorrhoeae* and MIP-1α and MIP-2 levels were measured 3 h post infection. N≥3, error bars represent SEM. One-Way ANOVA (with Tukey's post-test) was performed for relevant samples, *P<0.05, **P<0.01, ***P<0.001. Stars indicate significance against all other conditions.

Unlike WT PMNs, CEABAC neutrophils efficiently bind and phagocytose Opa-expressing *N. gonorrhoeae* (Figure 1C–D). This prompted us to consider whether uptake alone can account for the cytokine response of human CEACAM-expressing PMNs. To address this question, WT and CEABAC PMNs were pre-treated with cytochalasin D to inhibit phagocytosis prior to infection. While cytochalasin D pre-treatment led to a marked decrease in bacterial internalization, (Figure S1A), it had no effect on MIP-1α secretion (Figure 4A). Furthermore, we used PMNs from a transgenic mouse line expressing human CEACAM1 but no CEACAM3. While the human CEACAM1-expressing neutrophils can efficiently phagocytose Opa-expressing *N. gonorrhoeae* [43], the neutrophils showed little chemokine response to infection (Figure S1B).

In considering that reactive oxygen species (ROS) have been linked to the activation of various inflammatory signals [44], we also sought to confirm whether ROS formed during the well-characterized oxidative burst response to Opa-expressing *N. gonorrhoeae* [24], [25], [28], [41] explained the induced cytokine response. To this end, we prevented ROS production using the NADPH oxidase inhibitor diphenylene iodonium (DPI) and then measured cytokine production in WT and CEABAC neutrophils infected with Opa^−^ and Opa^+^ bacteria. While DPI completely abolished the otherwise high levels of ROS produced upon exposure of CEABAC PMNs to Opa^+^ *N. gonorrhoeae* (Figure S1C), this had no affect the chemokine response of CEABAC PMNs (Figure 4A). Taken together our data suggest that Opa protein-dependent engagement of CEACAM3 drives a cytokine response that is independent of its ability to promote bacterial phagocytosis and is not mediated by the ROS produced in response to infection.

### CEACAM-Opa interaction drives activation of NF-κB and MAPK signaling

A large proportion of genes identified in our gene array study (MIP-1α, MIP-2, TNF-α, IL-1α) are known to be activated by NF-κB. NF-κB transcription factors are major mediators of inflammation and have been shown to stimulate transcription of pro-inflammatory cytokines in response to LPS in PMNs [45] [46] [47]. NF-κB also governs transcriptional responses downstream of various ITAM receptors in other (non-neutrophil) cell types [48], including the innate immune receptor Dectin-1 [49]. This led us to investigate NF-κB activation downstream of CEACAM3. Consistent with NF-κB being a downstream effector of CEACAM3, IκBα was degraded more rapidly and completely in CEACAM-expressing PMNs than in the WT cells (Figure 4B).

The p38 mitogen associated kinase (MAPK) has been shown to act along side NF-κB in the activation of PMN transcriptional responses to LPS [50] [46]. Consequently, we considered whether p38 might also be involved in the CEACAM-mediated transcriptional response. Infected CEABAC neutrophils exhibit increased levels of p38 phosphorylation when compared to WT (Figure 4C), indicating that they are more effectively activated in response to *N. gonorrhoeae* infection. In contrast, the Erk1/2 MAPKs were not phosphorylated in a CEACAM-dependent manner (Figure 4D), suggesting selective activation of p38 kinase upon CEACAM ligation. To assess whether p38 activity contributed to the CEACAM-dependent cytokine response, PMNs were exposed to a p38-specific inhibitor (SB203580) prior to infection. This treatment effectively blocked production of MIP-1α and MIP-2 in CEABAC PMNs infected with Opa^+^ *N. gonorrhoeae* (Figure 4E), while inhibition of Erk1/2 phosphorylation had no effect on cytokine secretion (Figure S1D). Together, this data supports an essential role for p38 MAPK in the CEACAM-dependent transcriptional response.

**Figure 5:**
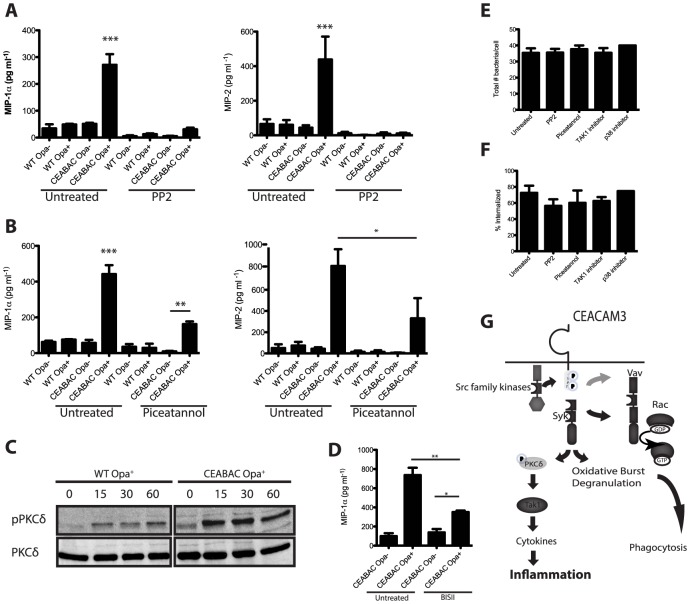
CEACAM3 signaling is required for the PMN cytokine response to *N. gonorrhoeae*. Inhibition of Src-family kinases and Syk leads to decreased cytokine production by infected PMNs. (**A, B**) WT and CEABAC PMNs were left untreated or pre-incubated with (**A**) Src-family kinase inhibitor (PP2, 10 µM), or (**B**) Syk inhibitor (piceatannol, 50 µM). PMNs were then infected with either non-opaque (Opa^−^) or Opa-expressing (Opa^+^) *N. gonorrhoeae*, and MIP-1α and MIP-2 levels were measured 3 h post infection. N≥3, error bars represent SEM. One-Way ANOVA was performed for relevant samples, *P<0.05, **P<0.01, ***P<0.001 (**C**) Opa^+^ *N. gonorrhoeae* infection leads to phosphorylation of PKCδ. WT and CEABAC PMNs were infected with Opa^+^ *N. gonorrhoeae* (MOI = 10 bacteria/PMN) for times indicated times. PKCδ activation was measured by immunoblot using phospho-PKCδ antibody. Immunoblot for PKCδ indicates equal protein loading. (**D**) CEABAC PMNs were left untreated or pre-incubated with PKC inhibitor (BIS II, 10 µM). PMNs were then infected with Opa^−^ or Opa^+^ *N. gonorrhoeae*, and MIP-1α levels were measured 3 h post infection. One-Way ANOVA (with Tukey's post-test) was performed for relevant samples, *P<0.05, **P<0.01, ***P<0.001. Unless otherwise indicated, stars indicate significance against all other conditions. (**E, F**) Inhibition of SFK, Syk, TAK1 or p38 does not affect bacterial binding (**E**) or phagocytosis (**F**). WT and CEABAC PMNs were left untreated or pre-incubated with indicated inhibitors. PMNs were then infected with Opa^+^ *N. gonorrhoeae* (MOI = 25) for 30 min. Intracellular and total bacteria were differentially stained, and quantified via immunofluorescence microscopy. (**G**) Schematic representation of proposed signaling pathway resulting in bacterial engulfment, activation of oxidative burst and degranulation, and cytokine production.

It has been reported that the mitogen-activated kinase kinase kinase (MAPKKK) family member TAK1 can activate both p38 MAPKs and NF-κB [51]. To test the involvement of TAK1 in the p38 activation observed during neisserial infection of PMNs, we used the TAK1 inhibitor (5z)-7-oxozeanol. TAK1 inhibition effectively abrogated MIP-1α and MIP-2 secretion by the Opa^+^ *N. gonorrhoeae*-infected CEABAC neutrophils (Figure 4F). Importantly, neither bacterial adherence nor phagocytosis by the PMNs were affected by either the p38 or TAK1 inhibitors (Figure 5E, F), confirming that the effect of these two compounds on cytokine expression was not due to their inhibition of these cellular processes.

### SFK, Syk, and PKCδ couple CEACAM3 to the downstream transcriptional response

CEABAC neutrophils express both human CEACAM3 and CEACAM6. Both receptors facilitate bacterial uptake, yet previous work has shown that the activation of neutrophil bactericidal processes, including degranulation and oxidative burst, occur via CEACAM3 alone [24], [25]. We sought to confirm whether the pro-inflammatory response observed in CEABAC neutrophils was solely mediated by CEACAM3, and to determine whether the inflammation depended upon the CEACAM3 ITAM-dependent signaling. Since CEACAM3, unlike the GPI-anchored CEACAM6, relies on phosphorylation of its cytoplasmic ITAM by Src family kinases (SFK) for its activation, we exploited the SFK-specific inhibitor PP2 that has previously been shown to block CEACAM3 ITAM-dependent signaling [22], [25], [52], [53]. Inhibition of SFK significantly abrogated cytokine production (Figure 5A), but did not affect bacterial adherence or phagocytosis (Figure 5E,F). Considered together, the data suggests that CEACAM3 ITAM phosphorylation is essential for induction of pro-inflammatory response. This also indicates a divergence in the bacterial engulfment and transcriptional pathways, since the tyrosine phosphorylation-independent CEACAM3- and CEACAM6-mediated engulfment of *N. gonorrhoeae* [24] is not, itself, sufficient to elicit the pro-inflammatory response. Work in other systems has shown that Syk kinase is an essential mediator of ITAM-mediated responses in general [54] and CEACAM3-specific neutrophil responses in particular [24], though there is some suggestion that CEACAM3 can signal independent of Syk [55]. Therefore, we tested whether Syk contributed to CEACAM3-dependent inflammatory signaling using the specific inhibitor, piceatannol. Inhibiting Syk function led to a significant reduction in chemokine production by CEABAC neutrophils (Figure 5B), implicating a critical role for this kinase in the CEACAM3-dependent pro-inflammatory cytokine responses.

Recently, the serine/threonine kinase PKCδ was shown to link signaling from the ITAM-containing innate immune receptor Dectin-1 to NF-κB activation in dendritic cells [56]. Consequently, we assessed PKCδ activation during *N. gonorrhoeae* infection of WT and/or CEABAC PMNs. CEABAC neutrophils showed substantially increased phosphorylation of PKCδ relative to that seen in WT PMNs (Figure 5C), suggesting it is also activated downstream of CEACAM3. Furthermore, pretreatment of CEABAC PMNs with the PKC inhibitor bisindolylmaleimide II (BIS II) led to the inhibition of MIP-1α production in response to Opa^+^ *N. gonorrhoeae* (Figure 5D), consistent with PKCδ actively contributing to the CEACAM3-driven inflammatory response. In summary, we have outlined a novel signaling cascade downstream of CEACAM3 that is distinct from CEACAM3-mediated bacterial engulfment and activation of antimicrobial responses (Figure 5G), and potentially contributes to the excessive inflammatory response typical of *N. gonorrhoeae* infection.

### CEACAM-Opa intensifies the inflammatory response *in vivo*

**Figure 6:**
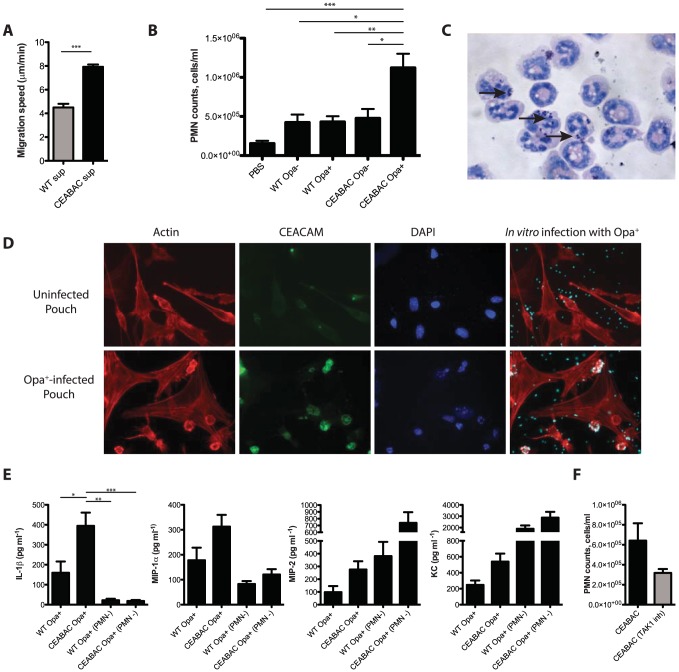
CEACAM binding stimulates the inflammatory response to *N. gonorrhoeae in vivo*. (**A**) Neutrophil migration assay. PMN migration speed towards *N. gonorrhoeae* infected CEABAC or WT neutrophil-derived supernatants was measured using a Zigmond chamber. One-Way ANOVA (with Tukey's post-test) was performed for relevant samples, ***P<0.001 (**B**) Neutrophil infiltration is more pronounced in human CEACAM-expressing mice in an infected subcutaneous air pouch model. Manual neutrophil counts of wash fluids collected from air pouches. ‘PBS’ denotes mice injected with sterile PBS. One-Way ANOVA was performed for relevant samples, *P<0.05, **P<0.01, ***P<0.001 (**C**) Giemsa-Wright stain of wash fluid collected from CEABAC air pouch infected with Opa^+^ *N. gonorrhoeae*. The arrows point towards *N. gonorrhoeae* inside the neutrophil. (**D**) Neutrophil-expressed CEACAMs mediate *N. gonorrhoeae* binding within the air pouch. Cells from trypsinized air pouches were grown on coverslips in the presence of antibiotics, and infected *in vitro* with Opa^+^ *N. gonorrhoeae*. Cells were visualized by staining for filamentous actin [16], CEACAMs (green), DNA (blue) and bacteria (cyan). (**E**) Levels of MIP-1α, MIP-2, KC, and IL-1β were measured in wash fluids collected from air pouches. PMN^−^ refers to mice in which neutrophils were depleted by administration of the Gr1-specific clone RB6-8C5 antibody one day prior to infection with Opa^+^ *N. gonorrhoeae*. One-Way ANOVA (with Tukey's post-test) was performed for relevant samples, *P<0.05, **P<0.01, ***P<0.001 (**F**) Inhibition of pro-inflammatory signaling reduces neutrophil infiltration into the air pouch. Neutrophil counts of wash fluids collected from CEABAC mice infected with Opa^+^ *N. gonorrhoeae* in the presence or absence of TAK1 inhibitor.

The heightened expression of pro-inflammatory cytokines by CEACAM-humanized PMNs should result in increased neutrophil chemotaxis to the site of *N. gonorrhoeae* infection, an outcome consistent with the clinical manifestations of gonorrhea. To test this, we collected supernatants from WT and CEABAC PMNs infected with Opa^+^ *N. gonorrhoeae*, and measured their ability to affect the speed and directionality of neutrophils using a Zigmond chamber. Consistent with the increased expression of chemotactic factors when CEABAC neutrophils are infected with *N. gonorrhoeae*, the chemotaxis of uninfected neutrophils was significantly greater in response to culture supernatants from the *N. gonorrhoeae*-infected CEABAC neutrophils, as compared to supernatants from infected WT neutrophils (Figure 6A). To understand the implications of these effects during *in vivo* infection, we measured the relative contribution of neutrophil CEACAM3 on inflammation using a subcutaneous air-pouch model, which allows the effective recovery and analysis of leukocyte recruitment to an otherwise sterile site [57]. Opa expression did not affect leukocyte recruitment into air pouches formed in WT mice. However, in CEABAC mice, the Opa-expressing gonococci elicited a significantly increased infiltration of neutrophils relative to that seen in response to Opa^−^ bacteria (Figure 6B). Giemsa-Wright staining of the CEABAC-Opa^+^ air pouch infiltrate showed that nearly all of the cells (>95%) present are PMNs (Figure 6C), with many neutrophils containing intracellular gonococci. Notably, the number of PMNs present in CEABAC mice infected with Opa^−^ bacteria reflected that seen in the WT mice, indicating that both Opa and human CEACAMs are required to drive the more intense inflammatory response.

Considering the marked increase in neutrophil response in the CEABAC animals, we considered whether it was possible that cells lining the air pouch could differentially associate with *N. gonorrhoeae*, which would contribute to the inflammatory milieu. To test this, we administered trypsin/EDTA into an uninfected air pouch from CEABAC mice, harvested the cells that were released, and then seeded them onto glass coverslips. The next day, cells were infected with Opa^+^ bacteria, and bacterial binding and CEACAM expression were assessed using immunofluorescence microscopy. Cells lining the pouch did not express CEACAM and did not bind or take up the *N. gonorrhoeae* (Figure 6D, top). When the same experiment was conducted on air pouches infected with Opa^+^ *N. gonorrhoeae*, we observed a large number of PMNs had infiltrated the air pouch lining. As expected, these PMNs expressed high levels of the human CEACAMs, and effectively bound the bacteria, unlike the adjacent fibroblast lining (Figure 6D, bottom). These results are consistent with the enhanced inflammation being due to differential association of the gonococci with the CEACAM3-expressing neutrophils, without obvious effect on their interaction with the surrounding tissues.

The air pouch experiments suggest that the CEACAM-dependent association between *N. gonorrhoeae* and the resident neutrophils promotes subsequent neutrophil recruitment, presumably due to the establishment of a chemotactic gradient. Consistent with this, the pro-inflammatory chemokines MIP-1α, MIP-2, KC, and IL-1β were all increased in the washes from Opa^+^-*N. gonorrhoeae* infected air pouches in CEABAC mice relative to that seen in the infected WT littermates (Figure 6E). To address whether neutrophils directly contribute to the higher levels of cytokines observed, we infected mice that had been depleted of neutrophils by administration of the Gr1-specific RB6-8C5 antibody prior to the introduction of *N. gonorrhoeae*. The levels of MIP-1α and IL-1β were significantly lower in the air pouches from PMN-depleted mice, indicating that neutrophils are the primary source for both of these chemokines (Figure 6E). Interestingly, the levels of KC and MIP-2 were dramatically higher in PMN-depleted mice, indicating that these chemokines are produced by cells other than PMNs under these conditions. While these chemokines are both produced by CEABAC neutrophils in response to *N. gonorrhoeae* (Figure 3A,B), they can also be produced by a variety of tissues [33], including synovial fibroblasts [58]. Considering that their levels increased upon neutrophil depletion, we interpret the increased response to suggest a delay in bacterial clearance in the absence of PMNs. The increased IL-1β and MIP-1α in CEABAC mice thereby reflect a local neutrophil response whereas MIP-2 and KC levels appear to be the cumulative effect of both neutrophil and underlying tissue responses.

To link the CEACAM3-dependent intracellular response evident from our cell-based experiments with inflammation *in vivo*, we repeated the air pouch experiment, this time assessing the effect of the TAK1 inhibitor, which was administered to mice 1 h prior to infecting the air pouch. Consistent with our model that the inhibition of CEACAM3 signaling would suppress the inflammatory response to *N. gonorrhoeae*, we observed a decrease in the number of infiltrating PMNs upon administration of the TAK1-inhibitor relative to the untreated animals (Figure 6F).

## Discussion

The picture of neutrophils as ever-ready weapons of defense aiming to achieve efficient pathogen clearance has become an axiom. While still true, recent evidence suggests that they have the ability to nuance their response through *de novo* gene expression in response to certain microbial cues. In addition to their classical role in direct microbial killing, neutrophils can produce a range of cytokines with the potential to affect inflammation through the activation and induced chemotaxis of various leukocytes [37], [59]. However, their specific contribution to the cytokine milieu and inflammatory response remains underappreciated *in vivo*. The emerging picture of neutrophils as a dynamic, responsive cell population has important implications for our understanding of the overzealous neutrophil response that typifies gonorrhea. Our findings reveal that the decoy receptor CEACAM3, in addition to facilitating the effective capture and killing of *N. gonorrhoeae*, also helps drive inflammation. While recruitment of more PMNs to combat infection would seem to be an effective innate immune strategy during early infection, the persistent exposure of CEACAM3-expressing PMNs to Opa-expressing gonococci can promote a self-perpetuating and, ultimately, pathogenic response such as is associated with gonorrhea or pelvic inflammatory disease.

**Figure 7:**
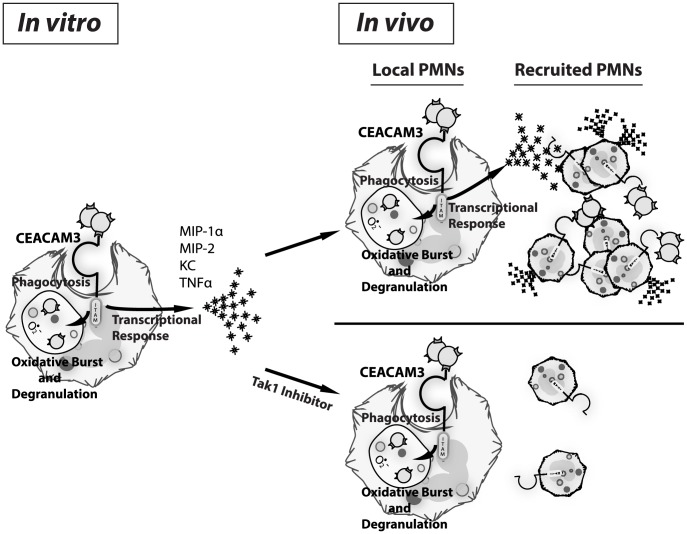
CEACAM3-mediated inflammation promotes immunopathology associated with *N. gonorrhoeae* infection. Upon *in vitro* infection, CEACAM3 allows efficient bacterial phagocytosis and clearance via activation of neutrophil antimicrobial responses including degranulation and oxidative burst. Concomitantly, CEACAM3 promotes *de novo* transcription of pro-inflammatory cytokines by the neutrophil (left panel). *In vivo*, this cytokine response recruits more neutrophils to the site of infection. As they arrive, these neutrophils will become activated via *N. gonorrhoeae* binding to CEACAM3, driving a self-propagating inflammatory process leading to the immunopathology associated with gonococcal disease (top right panel). By limiting the CEACAM3-dependent inflammatory cascade without affecting bacterial engulfment, such as via Tak1 inhibitor used in this study, bacterial clearance can continue while inflammation is reduced (bottom right panel).

In this work, we demonstrate that human CEACAM-expressing transgenic mouse PMNs respond to *N. gonorrhoeae* in a manner that parallels those of human PMNs. Unlike neutrophils from WT mice, the CEABAC neutrophils undergo a vigorous oxidative burst and degranulation response to *N. gonorrhoeae*. Since the pathology associated with gonococcal disease primarily arises due to tissue damage caused by the recruited neutrophils, we considered whether CEACAM-dependent interactions with the bacteria could also contribute to the inflammatory response. We observed that *N. gonorrhoeae* binding to human CEACAM3 leads to the acute activation of a pro-inflammatory transcriptional program that results in the production of the inflammatory mediators such as MIP-1α, MIP-2, KC and TNF-α. While relatively little is known about signal transduction in PMNs relative to other cell types, Opa binding to CEACAM3 elicits signaling via a pathway closely reminiscent of that triggered downstream of the innate anti-fungal receptor Dectin-1 [30], [60], [61]. As with Dectin-1, SFK and Syk kinase represent the first effectors downstream of CEACAM3, and their activity is required for the PMN oxidative burst and degranulation responses to Opa-expressing gonococci [24]. While Syk was shown to serve a regulatory role in the context of PMNs [62], we have observed that Syk contributes to the inflammatory cytokine response to *N. gonorrhoeae* by eliciting the PKCδ and TAK1-dependent activation of NF-κB. This CEACAM3-dependent expression of MIP-1α, MIP-2 and KC stimulates chemotaxis of uninfected neutrophils so as to augment their recruitment to the infected tissues, an effect that has the potential to contribute to both innate defense and the massive neutrophil recruitment that typifies gonorrhea (Figure 7).

The trade-off of having a direct link between CEACAM3 and inflammation is that, when uncontrolled, this response results in the ongoing infiltration of neutrophils, leading to permanent tissue damage such as is observed with *N. gonorrhoeae*-associated fallopian tube scarring and pelvic inflammatory disease. Supporting this model, we observed CEACAM-dependent increases in neutrophil recruitment into the gonococcal-infected tissues, brought on by the CEACAM3-dependent production of chemokines with the potential to promote continuous infiltration of neutrophils. Thus, CEACAM3 activation serves as a double-edged sword, promoting the immunopathology of gonorrhea through an over-activated immune response that is meant to clear the bacteria causing the infection. While the complete ablation of neutrophil recruitment would increase the bacterial burden, a better outcome would be to limit the chemokine response without blocking bacterial binding and phagocytosis (Figure 7). As a proof of concept for this, we used a TAK1 inhibitor *in vivo* to block the pro-inflammatory response and, thereby, the influx of neutrophils without affecting their phagocytic capacity. Satisfyingly, this treatment decreased PMN recruitment to that seen in WT mice, effectively eliminating the CEACAM3-dependent inflammation in response to *N. gonorrhoeae*.

It has been reported previously that the majority of neisserial isolates from infected patients have the capacity to bind to CEACAMs [5]. This may seem contradictory when considering the negative outcome for bacteria that bind to CEACAM3 on neutrophils. However, it is important to consider that, in contrast to CEACAM3, neisserial binding to other human CEACAMs facilitates attachment to mucosal epithelia [14], [63], [64], [65] and suppression of both innate [20] and adaptive [15], [16], [17] immune responses, activities which are central to the establishment and persistence of infection. In this respect, it is curious to contrast CEACAM1, which is the evolutionary precursor of the CEA family, with the evolutionarily ‘new’ CEACAM3. While CEACAM1 is present in all vertebrates and is broadly expressed on many cell types, CEACAM3 can only be found in humans and is only expressed by neutrophils. When this is considered along with the fact that CEACAM3 possesses no cell adhesion function yet has a sufficiently conserved extracellular domain that can be engaged by the neisserial Opa proteins, we and others have suggested that CEACAM3 functions as a decoy receptor that allows the capture and killing of CEACAM-targeting microbes. The apparent involvement of CEACAM3 in pro-inflammatory signaling establishes a role for neutrophils beyond basic microbial killing, and places CEACAM3 as both a potential contributor to the accelerated response to *N. gonorrhoeae* infection and, subsequently, to the immunopathology associated with the gonococcal disease. The evolutionary advent of CEACAM3 thus reflects the latest step in the ongoing dance between *Neisseria* and their only natural host, providing a snare that mobilizes our most potently bactericidal cells against this stealthy invader.

## Materials and Methods

### Ethics statement

All animal experiment procedures were approved by the Animal Ethics Review Committee of the University of Toronto (Approval #20010054 and #20010055), which is subject to the ethical and legal requirements under the province of Ontario's Animals for Research Act and the federal Council on Animal Care.

### Animals

Generation of the CEACAM1-humanized mouse line was previously described [43]. CEABAC2 mice, generated by stable integration of a human-derived bacterial artificial chromosome (BAC) encoding the human CEACAM3, CEACAM5, CEACAM6 and CEACAM7 genes, have been previously described [40]. Wild type (WT) mice used are littermates of the CEABAC2 animals.

### Reagents and antibodies

All reagents were from Sigma (Oakville, Ontario, Canada) unless otherwise indicated. The anti-gonococcal polyclonal rabbit antibody (UTR01) was described previously [18]. Rabbit anti- CEACAM polyclonal and normal serum was from Dako (Mississauga, ON). CEACAM pan-specific D14HD11, CEACAM1-specific 4/3/17 and CEACAM6-specific 9A6 antibodies were from Genovac (Freiburg, Germany), and the CEACAM3-specific Col-1 antibody was from Zymed (San Francisco, CA). Phospho-p38, p38, phospho-Erk1/2, Erk1/2, phospho-PKCδ and PKCδ-specific antibodies were form Cell Signaling Technology. The IκBα-specific antibody was from Santa Cruz Biotechnology (Santa Cruz, CA). Fluorescent conjugates were from Jackson ImmunoResearch Laboratories (Mississauga, ON), except Texas red-phalloidin, which was from Molecular Probes (Eugene, OR). The Tak1 inhibitor ((5z)-7-oxozeaenol) was from Millipore (Billerica, MA), and the p38 inhibitor (SB203580) and the Src family kinase-specific PP2 were from Calbiochem (La Jolla, CA). Erk1/2 inhibitor (UT0126) was purchased from Cell Signaling.

### Bacterial strains

The isogenic Opa^−^ and Opa^+^ (Opa_57_) *N. gonorrhoeae* MS11 strains (N302 and N313, respectively; [66]) were kindly provided by Dr. T.F. Meyer, and their phenotypes have been described previously [7].

### Primary neutrophil isolation

Human neutrophils were isolated from citrated whole blood taken from healthy volunteers by venipuncture using Ficoll-Paque Plus (Amersham Biosciences; Buckinghamshire, England). Contaminating erythrocytes were removed by dextran sedimentation and hypotonic shock, as described previously [29].

Mouse bone marrow neutrophils were taken from 8 to 10-week old mice that were humanely euthanized by CO_2_ inhalation. Femurs and tibias were removed, and bone marrow was isolated and separated on a discontinuous Percoll gradient (80%/65%/55%) as described previously by others [67]. Neutrophils were recovered at the 80%/65% interface.

### Human CEACAM expression in CEABAC neutrophils

WT and CEABAC neutrophils (10^6^ cells) were lysed in boiling SDS buffer and CEACAMs detected using SDS-PAGE and immunoblots probed with indicated CEACAM-specific antibodies. For flow cytometry analysis of cell-surface CEACAM expression, 10^6^ PMNs from CEABAC or WT littermates were spun down and fixed in 1% PFA in Hank's Buffered Saline Solution (HBSS) prior to immunofluorescence staining.

### Whole cell phosphorylation assays

10^6^ neutrophils per sample were infected with *N. gonorrhoeae* at multiplicity of infection [45] of 10 in 250 µl of HBSS. Infections were stopped by centrifugation at 2400 *g* for 3 min at 4°C, lysed in boiling SDS sample buffer, and boiled for a further 10 min. Samples were resolved by SDS-PAGE and immunoblotted.

### Bacterial infections for immunofluorescence microscopy

5×10^5^ WT or CEABAC bone marrow-derived PMNs were centrifuged onto washed mouse serum-coated coverslips at 1500 rpm for 10 min. Cells were infected at MOI of 25 (for binding and internalization studies) in a volume of 500 µl, re-centrifuged for 5 minutes at 500 rpm to facilitate bacterial association with cells, and then incubated at 37°C for indicated durations. Post-infection, samples were washed with HBSS, and fixed using 3.7% paraformaldehyde. Cells were stained for CEACAM, actin and bacteria and observed as described previously [6]. Intracellular bacteria were differentiated from extracellular via exclusion of antibody prior to membrane solubilization, as described [29].

### PMN killing assays

Killing assays were adapted from Ball et al. [41]. Briefly, adherent WT and CEABAC neutrophils were infected at an MOI = 1. At indicated time points, cells were washed and incubated with protease inhibitors for 15 minutes prior to lysis with 1% saponin and plated on GC agar. Bacterial survival was evaluated relative to CFUs present at 0 time point.

### Oxidative burst and degranulation assays

For chemiluminescence-based oxidative burst assay, 5×10^5^ cells were incubated with 25 µg/ml 5-amino-2,3-dihydro-1,4-phthalazinedione (‘luminol’; Sigma) in a volume of 100 µl, and then treated with agonists in a total volume of 200 µl, in triplicate. Infections proceeded for 60 min at 37°C, after which luminescence was read using a Tecan plate reader with i-control software. For flow cytometry-based degranulation assay (CD11b release), 10^6^ PMNs were treated with agonists in 500 µl of HBSS for 30 minutes at 37°C. Infections were stopped by centrifugation at 2,400 *g* for 3 min at RT and cell pellets then fixed in 1% PFA before staining with 1.25 µg of PE-conjugated rat anti-mouse CD11b in a total volume of 50 µl. Myeloperoxidase (MPO), elastase, and lactoferrin release assays were performed essentially as described by others. Briefly, 10^6^ PMNs were exposed to agonists in a total volume of 500 µl and then incubated for 30 minutes at 37°C. Cells were then pelleted and supernatants collected. For MPO assays, 50 µl of supernatant was mixed with 150 µl SureBlue tetramethylbenzidine peroxidase substrate (KPL; Gaithersberg, MD), and plates were read spectrophotometrically at 650 nm. For the elastase assay, 50 µl of supernatant was diluted 2-fold in PBS and incubated with 100 µl DQ elastin substrate conjugated to BODIPY FL (from the EnzCheck Elastase kit; Molecular Probes), and then incubated for 24 hours at RT before reading fluorescence with 488 nm excitation and 515 nm emission. For both MPO and elastase assays, a percentage (%) release is shown, calculated as the amount of the protein in the supernatant divided by the total amount in 10^6^ CHAPS-lysed cells.

Lactoferrin release from PMN granules was assayed by ELISA as described by others [48]. To induce release of primary granule components into medium, cells were pre-treated with 5 µM cytochalasin B for 5 minutes at 37°C prior to agonist treatment.

### PMN gene array experiments

CEABAC and WT bone marrow neutrophils (10^7^ cells) were either infected with Opa^+^ *N. gonorrhoeae* (MOI = 10) or left uninfected for 1 h. The infections were stopped by centrifugation at 2400 *g* for 5 min at 4°C. RNA was extracted and purified using the Qiagen RNeasy kit. Samples from 3 independent experiments were analyzed using an Illumina Mouse Whole Genome V2R2 array with 45,281 probes. The original data normalization and analysis were provided as a service by the Bioinformatics Department of the University Health Network (UHN) Microarray Centre, Toronto, ON. Data was analyzed using Genespring v11.0.1. 66 genes showed ≥2 fold change (FC) in gene expression in infected PMNs relative to uninfected controls. Gene lists were analyzed using the database for annotation, visualization, and integrated discovery (DAVID) (Huang da et al., 2009) and manual examination. To compare WT vs. CEABAC neutrophil responses, we considered genes ≥1.5 FC in CEABAC over WT.

### Cytokine measurements

10^6^ cells were infected with *N. gonorrhoeae* at MOI of 10 and incubated at 37°C for 3 h. Infections were then stopped by centrifugation at 2400 *g* for 5 min at 4°C, and supernatants were collected. Quantitative measurements of cytokines were performed using ELISA kits form R&D Systems (MIP-1α, KC and MIP-2) and BD Biosciences (TNF-α). For qRT-PCR, cells were infected as described above. At 3 h post infection, RNA was collected using Qiagen RNeasy kit and converted to cDNA using iScript RT Supermix (Bio-Rad Laboratories). qPCR was carried out using SsoAdvanced SYBR Green Supermix (Bio-Rad Laboratories). All transcript levels are shown as relative to those of GAPDH.

### Chemotaxis assay

Bone marrow neutrophils were isolated and suspended in HBSS with 1% gelatin (Sigma, G7041). A neutrophil suspension (1×10^6^/mL) was allowed to attach to bovine serum albumin (BSA, Sigma A7906; 1 mg/ml)-coated glass coverslips (22×40 mm, Fisher 12–543-A) at 37°C for 10 minutes. The coverslip was inverted into a Zigmond chamber (Neuroprobe, z02) and 100 µL HBSS media was added to the right chamber with 100 µL HBSS media mixed with supernatants derived from infected CEABAC or WT neutrophils added to the left chamber. Time-lapse video microscopy was used to examine neutrophil movements in Zigmond chambers. Images were captured at 20-second intervals with a Nikon Eclipse E1000 Microscope using the 40× objective. Cell-tracking software (Retrac version 2.1.01 Freeware) was used to characterize cellular chemotaxis from the captured images. Data comes from three independent experiments.

### Air pouch

CEABAC and WT littermate control mice (6–8 wk) were anesthetized with isoflurane, and dorsal air pouches were raised by injecting 3 ml sterile air subcutaneously on days 0 and a further 2 ml on day 3. On day 5, the mice were anesthetized with isoflurane and injected with 1 ml PBS containing 2×10^6^ cfu/ml of Opa^−^ or Opa^+^ *N. gonorrhoeae*. Mice were sacrificed 6 h after the injection, and air pouches were then washed with 2 ml PBS. The cells present in the wash were counted with a hemocytometer and analyzed by Diff-Quick staining of the cytospins. The supernatants were analyzed by ELISA, as described above. In some cases, air pouch fibroblasts were obtained by instilling 0.05% trypsin containing 0.5 mM EDTA in DMEM (3 ml/pouch), as previously described [68]. These cells were seeded onto glass coverslips in a 24-well plate, cultured in DMEM-10% FBS with antibiotics overnight, and then infected with Opa^+^ *N. gonorrhoeae* the next day for 30 min. Cells were fixed and stained for immunofluorescence microscopy.

### Neutrophil depletion

Neutrophil depletion from mice was achieved by a single i.p. injection of 200 µl of sterile saline containing 250 µg of the Gr-1 specific monoclonal antibody RB6-8C5 at 24 h prior to infection. The RB6-8C5 hybridoma was generously provided by Professor Paul Allen, Department of Pathology and Immunology, Washington University School of Medicine, St. Louis.

## Supporting Information

Figure S1 **Phagocytosis and production of reactive oxygen species are not essential for inflammatory cytokine production.** (**A**) Cytochalasin D treatment prevents bacterial uptake by CEABAC PMNs. WT and CEABAC PMNs were left untreated or pre-incubated with cytochalasin D (10 mg/ml) for 10 min prior to infection with either Opa^−^ or Opa^+^ *N. gonorrhoeae* (MOI 10). Extracellular and total bacteria were differentially stained, and quantified via immunofluorescence microscopy. (**B**) Human CEACAM1-expressing PMNs do not induce inflammatory cytokine production in response to Opa^+^ *N. gonorrhoeae*. Mouse (WT, CEABAC, and TG418) neutrophils were infected with either Opa^−^ or Opa^+^ *N. gonorrhoeae* (MOI 10). MIP-1α and MIP-2 was measured 3 h post infection. Data shown as fold induction by Opa^+^ infection over Opa^−^ infection. N = 3. (**C**) DPI treatment abolishes oxidative burst response in CEABAC PMNs infected with Opa^+^ *N. gonorrhoeae*. WT and CEABAC PMNs were left untreated or pre-incubated with DPI (10 µM) for 30 min prior to infection with either Opa^−^ or Opa^+^ *N. gonorrhoeae* (MOI 10). Oxidative burst was measured with as described in Materials and Methods. (**D**) Inhibition of Erk1/2 kinases does not affect inflammatory cytokine production. Erk1/2 inhibition does not affect cytokine production by CEABAC PMNs. WT and CEABAC PMNs were left untreated or pre-incubated with Erk1/2 inhibitor (UT0126, 10 µM) for 30 min prior to infection with Opa^+^ *N. gonorrhoeae* (MOI 10). MIP-1α production was measured 3 h post infection, N = 3.
(EPS)

## References

[1] World Health Organization DoRHaR (2012) Global action plan to control the spread and impact of antimicrobial resistance in *Neisseria gonorrhoeae* http://www.who.int/reproductivehealth/publications/rtis/9789241503501/en/

[2] World Health Organization DoRHaR (2012) Global incidence and prevalence of selected curable sexually transmitted infections - 2008. http://www.who.int/reproductivehealth/publications/rtis/stisestimates/en/

[3] Zhu W, Chen CJ, Thomas CE, Anderson JE, Jerse AE, et al (2011) Vaccines for gonorrhea: can we rise to the challenge? Frontiers in Microbiology 2: 124. 21687431 10.3389/fmicb.2011.00124 PMC3109613

[4] Biais N, Ladoux B, Higashi D, So M, Sheetz M (2008) Cooperative retraction of bundled type IV pili enables nanonewton force generation. PLoS Biology 6: e87. 18416602 10.1371/journal.pbio.0060087 PMC2292754

[5] Virji M, Makepeace K, Ferguson DJ, Watt SM (1996) Carcinoembryonic antigens (CD66) on epithelial cells and neutrophils are receptors for Opa proteins of pathogenic Neisseria. Molecular Microbiology 22: 941–950. 8971715 10.1046/j.1365-2958.1996.01551.x

[6] Gray-Owen SD, Dehio C, Haude A, Grunert F, Meyer TF (1997) CD66 carcinoembryonic antigens mediate interactions between Opa-expressing *Neisseria gonorrhoeae* and human polymorphonuclear phagocytes. The EMBO Journal 16: 3435–3445. 9218786 10.1093/emboj/16.12.3435 PMC1169969

[7] Gray-Owen SD, Lorenzen DR, Haude A, Meyer TF, Dehio C (1997) Differential Opa specificities for CD66 receptors influence tissue interactions and cellular response to *Neisseria gonorrhoeae* . Molecular Microbiology 26: 971–980. 9426134 10.1046/j.1365-2958.1997.6342006.x

[8] Chen T, Grunert F, Medina-Marino A, Gotschlich EC (1997) Several carcinoembryonic antigens (CD66) serve as receptors for gonococcal opacity proteins. The Journal of Experimental Medicine 185: 1557–1564. 9151893 10.1084/jem.185.9.1557 PMC2196295

[9] Bos MP, Grunert F, Belland RJ (1997) Differential recognition of members of the carcinoembryonic antigen family by Opa variants of *Neisseria gonorrhoeae* . Infection and Immunity 65: 2353–2361. 9169774 10.1128/iai.65.6.2353-2361.1997 PMC175326

[10] Virji M, Evans D, Hadfield A, Grunert F, Teixeira AM, et al (1999) Critical determinants of host receptor targeting by *Neisseria meningitidis* and *Neisseria gonorrhoeae*: identification of Opa adhesiotopes on the N-domain of CD66 molecules. Molecular Microbiology 34: 538–551. 10564495 10.1046/j.1365-2958.1999.01620.x

[11] Billker O, Popp A, Gray-Owen SD, Meyer TF (2000) The structural basis of CEACAM-receptor targeting by neisserial Opa proteins. Trends in Microbiology 8: 258–260 discussion 260–251. 10838580 10.1016/s0966-842x(00)01771-6

[12] Popp A, Dehio C, Grunert F, Meyer TF, Gray-Owen SD (1999) Molecular analysis of neisserial Opa protein interactions with the CEA family of receptors: identification of determinants contributing to the differential specificities of binding. Cellular Microbiology 1: 169–181. 11207550 10.1046/j.1462-5822.1999.00017.x

[13] McGee ZA, Stephens DS, Hoffman LH, Schlech WF 3rd, Horn RG (1983) Mechanisms of mucosal invasion by pathogenic Neisseria. Reviews of Infectious Diseases 5 Suppl 4: S708–714. 6415784 10.1093/clinids/5.supplement_4.s708

[14] Wang J, Gray-Owen SD, Knorre A, Meyer TF, Dehio C (1998) Opa binding to cellular CD66 receptors mediates the transcellular traversal of *Neisseria gonorrhoeae* across polarized T84 epithelial cell monolayers. Molecular Microbiology 30: 657–671. 9822830 10.1046/j.1365-2958.1998.01102.x

[15] Boulton IC, Gray-Owen SD (2002) Neisserial binding to CEACAM1 arrests the activation and proliferation of CD4+ T lymphocytes. Nature Immunology 3: 229–236. 11850628 10.1038/ni769

[16] Lee HS, Boulton IC, Reddin K, Wong H, Halliwell D, et al (2007) Neisserial outer membrane vesicles bind the coinhibitory receptor carcinoembryonic antigen-related cellular adhesion molecule 1 and suppress CD4+ T lymphocyte function. Infection and Immunity 75: 4449–4455. 17620353 10.1128/IAI.00222-07 PMC1951172

[17] Lee HS, Ostrowski MA, Gray-Owen SD (2008) CEACAM1 dynamics during *Neisseria gonorrhoeae* suppression of CD4+ T lymphocyte activation. Journal of Immunology 180: 6827–6835. 10.4049/jimmunol.180.10.6827 18453603

[18] Pantelic M, Kim YJ, Bolland S, Chen I, Shively J, et al (2005) *Neisseria gonorrhoeae* kills carcinoembryonic antigen-related cellular adhesion molecule 1 (CD66a)-expressing human B cells and inhibits antibody production. Infection and Immunity 73: 4171–4179. 15972507 10.1128/IAI.73.7.4171-4179.2005 PMC1168567

[19] Yu Q, Chow EM, McCaw SE, Hu N, Byrd D, et al (2013) Association of *Neisseria gonorrhoeae* Opa(CEA) with dendritic cells suppresses their ability to elicit an HIV-1-specific T cell memory response. PloS One 8: e56705. 23424672 10.1371/journal.pone.0056705 PMC3570455

[20] Slevogt H, Zabel S, Opitz B, Hocke A, Eitel J, et al (2008) CEACAM1 inhibits Toll-like receptor 2-triggered antibacterial responses of human pulmonary epithelial cells. Nature Immunology 9: 1270–1278. 18836450 10.1038/ni.1661

[21] Gray-Owen SD, Blumberg RS (2006) CEACAM1: contact-dependent control of immunity. Nature Reviews Immunology 6: 433–446. 10.1038/nri1864 16724098

[22] Schmitter T, Agerer F, Peterson L, Munzner P, Hauck CR (2004) Granulocyte CEACAM3 is a phagocytic receptor of the innate immune system that mediates recognition and elimination of human-specific pathogens. The Journal of Experimental Medicine 199: 35–46. 14707113 10.1084/jem.20030204 PMC1887732

[23] Pils S, Gerrard DT, Meyer A, Hauck CR (2008) CEACAM3: an innate immune receptor directed against human-restricted bacterial pathogens. International Journal of Medical Microbiology 298: 553–560. 18606569 10.1016/j.ijmm.2008.04.005

[24] Sarantis H, Gray-Owen SD (2012) Defining the roles of human carcinoembryonic antigen-related cellular adhesion molecules during neutrophil responses to *Neisseria gonorrhoeae* . Infection and Immunity 80: 345–358. 22064717 10.1128/IAI.05702-11 PMC3255650

[25] Sarantis H, Gray-Owen SD (2007) The specific innate immune receptor CEACAM3 triggers neutrophil bactericidal activities via a Syk kinase-dependent pathway. Cellular Microbiology 9: 2167–2180. 17506820 10.1111/j.1462-5822.2007.00947.x

[26] Booth JW, Telio D, Liao EH, McCaw SE, Matsuo T, et al (2003) Phosphatidylinositol 3-kinases in carcinoembryonic antigen-related cellular adhesion molecule-mediated internalization of *Neisseria gonorrhoeae* . The Journal of Biological Chemistry 278: 14037–14045. 12571236 10.1074/jbc.M211879200

[27] Buntru A, Kopp K, Voges M, Frank R, Bachmann V, et al (2011) Phosphatidylinositol 3′-kinase activity is critical for initiating the oxidative burst and bacterial destruction during CEACAM3-mediated phagocytosis. The Journal of Biological Chemistry 286: 9555–9566. 21216968 10.1074/jbc.M110.216085 PMC3059060

[28] Smirnov A, Daily KP, Criss AK (2014) Assembly of NADPH Oxidase in Human Neutrophils Is Modulated by the Opacity-Associated Protein Expression State of *Neisseria gonorrhoeae* . Infection and Immunity 82: 1036–1044. 24343654 10.1128/IAI.00881-13 PMC3957997

[29] McCaw SE, Schneider J, Liao EH, Zimmermann W, Gray-Owen SD (2003) Immunoreceptor tyrosine-based activation motif phosphorylation during engulfment of *Neisseria gonorrhoeae* by the neutrophil-restricted CEACAM3 (CD66d) receptor. Molecular Microbiology 49: 623–637. 12864848 10.1046/j.1365-2958.2003.03591.x

[30] Buntru A, Roth A, Nyffenegger-Jann NJ, Hauck CR (2012) HemITAM signaling by CEACAM3, a human granulocyte receptor recognizing bacterial pathogens. Archives of Biochemistry and Biophysics 524: 77–83. 22469950 10.1016/j.abb.2012.03.020

[31] Sadarangani M, Pollard AJ, Gray-Owen SD (2011) Opa proteins and CEACAMs: pathways of immune engagement for pathogenic Neisseria. FEMS Microbiology Reviews 35: 498–514. 21204865 10.1111/j.1574-6976.2010.00260.x

[32] Bazzoni F, Cassatella MA, Rossi F, Ceska M, Dewald B, et al (1991) Phagocytosing neutrophils produce and release high amounts of the neutrophil-activating peptide 1/interleukin 8. The Journal of Experimental Medicine 173: 771–774. 1997655 10.1084/jem.173.3.771 PMC2118810

[33] Kolaczkowska E, Kubes P (2013) Neutrophil recruitment and function in health and inflammation. Nature Reviews Immunology 13: 159–175. 10.1038/nri3399 23435331

[34] Jaillon S, Galdiero MR, Del Prete D, Cassatella MA, Garlanda C, et al (2013) Neutrophils in innate and adaptive immunity. Seminars in Immunopathology 35: 377–394. 23553214 10.1007/s00281-013-0374-8

[35] Subrahmanyam YV, Yamaga S, Prashar Y, Lee HH, Hoe NP, et al (2001) RNA expression patterns change dramatically in human neutrophils exposed to bacteria. Blood 97: 2457–2468. 11290611 10.1182/blood.v97.8.2457

[36] Kobayashi SD, Voyich JM, Buhl CL, Stahl RM, DeLeo FR (2002) Global changes in gene expression by human polymorphonuclear leukocytes during receptor-mediated phagocytosis: cell fate is regulated at the level of gene expression. Proceedings of the National Academy of Sciences of the United States of America 99: 6901–6906. 11983860 10.1073/pnas.092148299 PMC124501

[37] Scapini P, Lapinet-Vera JA, Gasperini S, Calzetti F, Bazzoni F, et al (2000) The neutrophil as a cellular source of chemokines. Immunological Reviews 177: 195–203. 11138776 10.1034/j.1600-065x.2000.17706.x

[38] Kammerer R, Popp T, Hartle S, Singer BB, Zimmermann W (2007) Species-specific evolution of immune receptor tyrosine based activation motif-containing CEACAM1-related immune receptors in the dog. BMC Evolutionary Biology 7: 196. 17945019 10.1186/1471-2148-7-196 PMC2110893

[39] Voges M, Bachmann V, Kammerer R, Gophna U, Hauck CR (2010) CEACAM1 recognition by bacterial pathogens is species-specific. BMC Microbiology 10: 117. 20406467 10.1186/1471-2180-10-117 PMC2871271

[40] Chan CH, Stanners CP (2004) Novel mouse model for carcinoembryonic antigen-based therapy. Molecular Therapy : the Journal of the American Society of Gene Therapy 9: 775–785. 15194045 10.1016/j.ymthe.2004.03.009

[41] Ball LM, Criss AK (2013) Constitutively Opa-expressing and Opa-deficient *Neisseria gonorrhoeae* strains differentially stimulate and survive exposure to human neutrophils. Journal of Bacteriology 195: 2982–2990. 23625842 10.1128/JB.00171-13 PMC3697530

[42] Huang da W, Sherman BT, Lempicki RA (2009) Systematic and integrative analysis of large gene lists using DAVID bioinformatics resources. Nature Protocols 4: 44–57. 19131956 10.1038/nprot.2008.211

[43] Gu A, Zhang Z, Zhang N, Tsark W, Shively JE (2010) Generation of human CEACAM1 transgenic mice and binding of Neisseria Opa protein to their neutrophils. PloS One 5: e10067. 20404914 10.1371/journal.pone.0010067 PMC2852402

[44] Tamas D, Cecilia C, Sanae Ben M, Thilo MF, Siegfried W, et al (2010) Potentiation of Epithelial Innate Host Responses by Intercellular Communication. PLoS Pathogens 10.1371/journal.ppat.1001194 PMC2987820 21124989

[45] Tamassia N, Le Moigne V, Calzetti F, Donini M, Gasperini S, et al (2007) The MyD88-independent pathway is not mobilized in human neutrophils stimulated via TLR4. Journal of Immunology 178: 7344–7356. 10.4049/jimmunol.178.11.7344 17513785

[46] Cloutier A, Ear T, Blais-Charron E, Dubois CM, McDonald PP (2007) Differential involvement of NF-kappaB and MAP kinase pathways in the generation of inflammatory cytokines by human neutrophils. Journal of leukocyte biology 81: 567–577. 17062602 10.1189/jlb.0806536

[47] McDonald PP, Bald A, Cassatella MA (1997) Activation of the NF-kappaB pathway by inflammatory stimuli in human neutrophils. Blood 89: 3421–3433. 9129050

[48] Abram CL, Lowell CA (2007) The expanding role for ITAM-based signaling pathways in immune cells. Science's STKE : signal transduction knowledge environment 2007: re2. 10.1126/stke.3772007re2 17356173

[49] Kingeter LM, Lin X (2012) C-type lectin receptor-induced NF-kappaB activation in innate immune and inflammatory responses. Cellular & molecular immunology 9: 105–112. 22246129 10.1038/cmi.2011.58 PMC4002809

[50] Fessler MB, Malcolm KC, Duncan MW, Worthen GS (2002) A genomic and proteomic analysis of activation of the human neutrophil by lipopolysaccharide and its mediation by p38 mitogen-activated protein kinase. The Journal of Biological Chemistry 277: 31291–31302. 11943771 10.1074/jbc.M200755200

[51] Landstrom M (2010) The TAK1-TRAF6 signalling pathway. The international Journal of Biochemistry & Cell Biology 42: 585–589. 20060931 10.1016/j.biocel.2009.12.023

[52] McCaw SE, Liao EH, Gray-Owen SD (2004) Engulfment of *Neisseria gonorrhoeae*: revealing distinct processes of bacterial entry by individual carcinoembryonic antigen-related cellular adhesion molecule family receptors. Infection and Immunity 72: 2742–2752. 15102784 10.1128/IAI.72.5.2742-2752.2004 PMC387857

[53] Hauck CR, Meyer TF, Lang F, Gulbins E (1998) CD66-mediated phagocytosis of Opa52 *Neisseria gonorrhoeae* requires a Src-like tyrosine kinase- and Rac1-dependent signalling pathway. The EMBO Journal 17: 443–454. 9430636 10.1093/emboj/17.2.443 PMC1170395

[54] Mocsai A, Ruland J, Tybulewicz VL (2010) The SYK tyrosine kinase: a crucial player in diverse biological functions. Nature Reviews Immunology 10: 387–402. 10.1038/nri2765 PMC4782221 20467426

[55] Schmitter T, Pils S, Sakk V, Frank R, Fischer KD, et al (2007) The granulocyte receptor carcinoembryonic antigen-related cell adhesion molecule 3 (CEACAM3) directly associates with Vav to promote phagocytosis of human pathogens. Journal of immunology 178: 3797–3805. 10.4049/jimmunol.178.6.3797 17339478

[56] Strasser D, Neumann K, Bergmann H, Marakalala MJ, Guler R, et al (2012) Syk kinase-coupled C-type lectin receptors engage protein kinase C-sigma to elicit Card9 adaptor-mediated innate immunity. Immunity 36: 32–42. 22265677 10.1016/j.immuni.2011.11.015 PMC3477316

[57] Edwards JC, Sedgwick AD, Willoughby DA (1981) The formation of a structure with the features of synovial lining by subcutaneous injection of air: an *in vivo* tissue culture system. The Journal of Pathology 134: 147–156. 7019400 10.1002/path.1711340205

[58] Pierer M, Rethage J, Seibl R, Lauener R, Brentano F, et al (2004) Chemokine secretion of rheumatoid arthritis synovial fibroblasts stimulated by Toll-like receptor 2 ligands. Journal of Immunology 172: 1256–1265. 10.4049/jimmunol.172.2.1256 14707104

[59] Tateda K, Moore TA, Deng JC, Newstead MW, Zeng X, et al (2001) Early recruitment of neutrophils determines subsequent T1/T2 host responses in a murine model of *Legionella pneumophila* pneumonia. Journal of Immunology 166: 3355–3361. 10.4049/jimmunol.166.5.3355 11207291

[60] Kerrigan AM, Brown GD (2010) Syk-coupled C-type lectin receptors that mediate cellular activation via single tyrosine based activation motifs. Immunological Reviews 234: 335–352. 20193029 10.1111/j.0105-2896.2009.00882.x

[61] Goodridge HS, Underhill DM, Touret N (2012) Mechanisms of Fc receptor and Dectin-1 activation for phagocytosis. Traffic 13: 1062–1071. 22624959 10.1111/j.1600-0854.2012.01382.x

[62] Zhang X, Majlessi L, Deriaud E, Leclerc C, Lo-Man R (2009) Coactivation of Syk kinase and MyD88 adaptor protein pathways by bacteria promotes regulatory properties of neutrophils. Immunity 31: 761–771. 19913447 10.1016/j.immuni.2009.09.016

[63] Muenzner P, Bachmann V, Zimmermann W, Hentschel J, Hauck CR (2010) Human-restricted bacterial pathogens block shedding of epithelial cells by stimulating integrin activation. Science 329: 1197–1201. 20813953 10.1126/science.1190892

[64] Muenzner P, Rohde M, Kneitz S, Hauck CR (2005) CEACAM engagement by human pathogens enhances cell adhesion and counteracts bacteria-induced detachment of epithelial cells. The Journal of Cell Biology 170: 825–836. 16115956 10.1083/jcb.200412151 PMC2171332

[65] Johswich KO, McCaw SE, Islam E, Sintsova A, Gu A, et al (2013) *In vivo* adaptation and persistence of *Neisseria meningitidis* within the nasopharyngeal mucosa. PLoS Pathogens 9: e1003509. 23935487 10.1371/journal.ppat.1003509 PMC3723569

[66] Kupsch EM, Knepper B, Kuroki T, Heuer I, Meyer TF (1993) Variable opacity (Opa) outer membrane proteins account for the cell tropisms displayed by *Neisseria gonorrhoeae* for human leukocytes and epithelial cells. The EMBO Journal 12: 641–650. 8440254 10.1002/j.1460-2075.1993.tb05697.x PMC413248

[67] Sun CX, Downey GP, Zhu F, Koh AL, Thang H, et al (2004) Rac1 is the small GTPase responsible for regulating the neutrophil chemotaxis compass. Blood 104: 3758–3765. 15308574 10.1182/blood-2004-03-0781

[68] Kukulski F, Ben Yebdri F, Bahrami F, Levesque SA, Martin-Satue M, et al (2010) The P2 receptor antagonist PPADS abrogates LPS-induced neutrophil migration in the murine air pouch via inhibition of MIP-2 and KC production. Molecular Immunology 47: 833–839. 19889460 10.1016/j.molimm.2009.09.037 PMC5142838

